# Normal Development of Local Neurovascular Interactions and the Diagnostic Value of Resting State Functional MRI in Neurovascular Deficiency Based on the Example of Neonatal Anesthesia Exposure

**DOI:** 10.3389/fneur.2021.664706

**Published:** 2021-04-29

**Authors:** Daniil P. Aksenov

**Affiliations:** ^1^Department of Radiology, NorthShore University HealthSystem, Evanston, IL, United States; ^2^Department of Anesthesiology, NorthShore University HealthSystem, Evanston, IL, United States

**Keywords:** toxicity, infant, neurovascular coupling, rsfmr imaging, vasomotion, sevoflurane

## Introduction

Anesthesia-induced learning deficiency is a serious problem in pediatrics. However, it is not clear which instrumental methods can be used for the evaluation of anesthesia-induced toxicity and for the prediction of behavioral and learning deficiencies. Here, we discuss the diagnostic and prognostic role of resting state fMRI and possible mechanisms which are responsible for anesthesia-induced neurovascular deficiency.

Many millions of people, including 6 million children, undergo general anesthesia each year during the course of surgery, imaging, and other medical procedures (1). The potential pathogenic effects of anesthesia on the developing brain have become increasingly concerning (2–8). For example, it was found that adolescents who underwent anesthesia as young children were more than twice as likely to display behavioral disorders or developmental deficits, including learning disabilities, autism, and speech impediments (9).

The pathological effects reported in humans are supported by a number of animal studies that have sought to elucidate the possible mechanisms that could explain the anesthesia-related deficits in learning and behavior. It was found that neonatal exposure to commonly used anesthetics leads to apoptotic neurodegeneration in the developing brain, which involves such structures as the cerebral cortex, hippocampus, thalamus, etc. (10, 11). Learning performance, including classical conditioning, measured at adolescence or adult age, was also affected by neonatal anesthesia exposure (12–14). However, it is not clear which instrumental methods can be used to evaluate these impairments. We suggest that neonatal widespread apoptotic neurodegeneration should affect further development, specifically as a manifestation of deficiency in neurovascular interactions, and this effect can be visualized using functional MRI (fMRI).

There are two major types of fMRI which can be used in patients: stimulus (task) evoked and resting state (rsfMRI). The first type can identify brain regions which respond to stimulation (visual, somatosensory, etc.) or are involved in a specific task performance, whereas resting state fMRI can visualize spontaneous activity of local and global brain networks. Generally, rsfMRI is preferable due to its simplicity and short duration, but stimulus or task evoked fMRI is applicable when brain pathology is localized and the function of the affected brain region can be easily tested. Anesthesia-induced pathology is not typically associated with obvious neurological deficit which can be revealed with a standard examination by a pediatric neurologist (15), and, thus, regional rsfMRI seems to be more useful because it represents basic neurovascular interactions in local networks. We will review it in greater detail in the next section.

## The Possible Relationship Between Anesthesia-Induced Apoptotic Neurodegeneration, Neurovascular Interactions, and FMRI

fMRI reflects the dynamics of oxy/deoxyhemoglobin (16) and, thus, directly depends on arteriolar vasomotion, which can be defined as a change of the arteriolar diameter either due to intrinsic vascular mechanisms or in response to alterations of neuronal activity. It was shown that stimulus-evoked vasomotion can be driven by both the excitatory glutamatergic (17) and the inhibitory GABAergic systems (18–20). However, the role of excitatory and inhibitory systems for resting state vasomotion is not clear. Glutamate-ergic excitatory mechanisms include nitric oxide (NO), the vasoactive metabolites of arachidonic acid (AA), and K+ (21, 22). A number of metabolites of AA are vasoactive (23, 24) and act on vessels, for example, by activating K+ channels (25) or prostanoid receptors (26). AA metabolites are primarily synthetized in astrocytes (27), and it has been proposed that neuronal activity produces an increase of Ca2+ in astrocytes, thereby causing vasodilation. An increase in extracellular potassium ions (K+) levels can also dilate vessels, and so the astrocytic K+ siphoning mechanism has also been suggested as a mechanism for neurovascular coupling (22, 28). Moreover, resting state vasomotion can depend mainly on the function of GABAergic interneurons which control it directly through GABA receptors on arterioles (29–31). Since neonatal anesthesia causes massive apoptotic neurodegeneration, the development of glia, glutamatergic, and GABAergic systems can also be affected (11, 32–34).

Arteriolar vasomotion follows a typical developmental trajectory. In adults, it is characterized by a 10 cycles per minute (cpm) dominant power frequency (35, 36), whereas underdeveloped vasomotion has a dominant frequency below 3 cpm (37). This shift in frequency of vasomotion and corresponding brain tissue oxygen oscillations represents a normal development and is needed to prevent localized hypoxia depending on the oxygen demand by developing neurons and neuronal networks (36). If neonatal anesthesia affects the development of the glutamatergic system, GABAergic system, or normal formation of astrocytes, the development of vasomotion can be affected as well. Specifically, resting state vasomotion may not obtain mature properties in time or may simply never obtain them to the full extent. This will be visualized by resting state fMRI (rsfMRI), and short-range connectivity will show clear changes corresponding to the local nature of the described mechanisms. Indeed, there is an initial study confirming this scenario, which reported the deficiency in short-range rsfMRI connectivity in adult animals after neonatal anesthesia exposure (38). It was found that the amplitude of regional low-frequency fluctuation (ALFF) was significantly higher in anesthesia-exposed rabbits in the somatosensory cortices, and ALFF in those areas could be a significant predictor of the learning performance for eyeblink conditioning responses.

## Discussion

rsfMRI is a potentially important instrument to evaluate neurovascular interactions after neonatal anesthesia exposure. This method requires minimal subject cooperation, has a short run-time, and can be done almost in any human age. If the state of neurovascular interactions is known, it can help to indirectly evaluate the neuronal damage induced by anesthetics and predict later-developing behavioral and learning deficiencies. When the neuronal mechanisms of rsfMRI become more evident, rsfMRI will be an even more valuable tool for estimation of damage in specific neuronal or glial subpopulations and the development of proper treatment strategies, which may precede the manifestation of anesthesia-induced learning or behavioral deficiency.

rsfMRI has several serious procedural limitations, which can affect the results and their interpretations. One limitation is the usage of anesthesia during MR imaging. It was shown previously that even a small concentration (0.25 MAC) of a general anesthetic affects both neuronal activity and the BOLD fMRI response in such a way as to significantly alter their previous relationship in awake state (39). A larger concentration of a general anesthetic can greatly decrease or even completely abolish BOLD fMRI signal (40). Moreover, it seems that this effect might depend on the type of general anesthetic because, for example, the level of suppression of neuronal activity is very different under 1 MAC of sevoflurane and 1 MAC of isoflurane (41). Typically, local resting state activity of a majority of neurons is not visibly synchronized in the awake state. However, neuronal activity can be partially synchronized under anesthesia (42, 43). Independently of rsfMRI mechanisms, neuronal synchronization can induce an additional factor of correlation between rsfMRI and neuronal activity because both the oxygen delivery and consumption curves may be synchronized with simultaneous firing of multiple neurons. Most importantly, it was shown that anesthesia directly affects vasomotion in adults by shifting the power spectrum of brain tissue oxygen oscillations to the left (35) which mimics neonatal vasomotion frequency and can create a misleading impression about the neurovascular deficiency. However, the fact that cerebral blood flow in the awake state is consistently different from the anesthetized state, might also have a positive physiological meaning because it indirectly supports the hypothesis about neuronal mechanisms of rsfMRI. Neuronal activity suppressed by anesthesia is followed by altered rsfMRI signal, which indicates the dependence between these two phenomena. If to imagine a scenario when neuronal activity decreases but rsfMRI does not change, it would mean that rsfMRI does not rely on the function of neurons. Thus, if neuronal apoptosis, associated with neonatal anesthesia, leads to neurovascular deficiency, this can be revealed by rsfMRI based on the markers of frequency, amplitude, periodicity, or synchrony of arteriolar vasomotion.

It is well-known that anesthesia is typically used during MR imaging in young children. MR is very sensitive to physical motions and sometimes it is hard to reach the necessary level of cooperation with a child, particularly if it is an infant. Also, children might express a fear of being inside the imager or could be distracted or even stressed by the loud noise. In spite of that we insist that anesthesia should be avoided during fMRI because the informativeness of the obtained data and the usefulness of the procedure itself will be questionable. Moreover, if we speak about the side effects of anesthesia in children, additional anesthesia exposure at a young age may have a superimposed negative effect and worsen behavioral outcome. Indeed, it was shown that a single exposure to anesthesia for a relatively short time period (i.e., once for 2 h) has much less of a neurotoxic effect than multiple exposures including the usage of different anesthetics (44, 45). However, there are two possible solutions how to avoid the use of anesthesia during fMRI. The first one is to image young children during normal sleep. This method was previously described (46) and, taking into account that the duration of rsfMRI can be only several minutes, this approach is feasible. The second solution is to image children who are old enough to cooperate.

That is why another important aspect of rsfMRI application after neonatal anesthesia is which minimal age should be chosen for this test. The typical human age range vulnerable to anesthesia is up to 4–5 years postnatal (47). If neonates or young children (up to 1 year postnatal) were exposed to anesthesia, the choice of the first rsfMRI time point is difficult, because it directly depends on the mechanisms of rsfMRI. Specifically, it depends on the development of substance which drives vasomotion. Since it can be glutamatergic, GABAergic, glial systems, or a combination of them, and because their developmental curves are different, the data obtained during the first few years of life should be analyzed taking into account that diagnostically valuable maturation of neurovascular coupling has not yet occurred. For example, the postnatal development of glutamatergic system undergoes several stages when the expression of different glutamate proteins can reach its peak at 1 year postnatal (GluN1) or even at 5–11 years (GluA2) (48) and the intensive growth of short-range axons occurs during first 5 years of life (49). Moreover, the development of short-range connections is not linear. Multiple studies reported an inverted U-shaped curve of short-range connectivity during development from the newborn age to the adulthood (49). The development of neurovascular coupling should reflect the development of neuronal system; even neurovascular responses to stimulation in children exceeding 36 months are different from adult responses (50). We suggest that during first years of human life, arteriolar vasomotion cannot be used as a diagnostic or prognostic factor since its frequency and amplitude will be still at the underdeveloped level and no difference will be found between children exposed and unexposed to anesthesia. Thus, the time of the first rsfMRI session after neonatal anesthesia exposure should be greatly delayed until a child will reach at least 3 years of age.

The choice of specific brain structures for rsfMRI again depends on the time factor. The sensory systems develop typically faster than learning-related systems [for review see (51)] and arteriolar vasomotion was extensively studied mostly in cerebral cortex. That is why we suggest using sensory cortical areas (for example, somatosensory or visual) as regions of interest (ROI). Note that sensory systems are more resistant to damage if to compare them with learning-related systems (51) due to the high compensatory abilities of sensory systems at the level of the neuronal network and the processing of sensory information; however, neurovascular interactions may not have such advanced level of adaptation.

In conclusion, we recommend using rsfMRI as a method to indirectly evaluate anesthesia-induced effects in children and predict learning deficiency. However, the factor of age and development of neurovascular coupling should be considered for short-range rsfMRI.

## Author Contributions

DA wrote this manuscript.

## Conflict of Interest

The author declares that the research was conducted in the absence of any commercial or financial relationships that could be construed as a potential conflict of interest.
